# Benzyl Isothiocyanate Induces Apoptosis and Inhibits Tumor Growth in Canine Mammary Carcinoma via Downregulation of the Cyclin B1/Cdk1 Pathway

**DOI:** 10.3389/fvets.2020.580530

**Published:** 2020-11-11

**Authors:** Nan Cheng, Hongxiu Diao, Zhaoyan Lin, Jiafeng Gao, Ying Zhao, Weijiao Zhang, Qi Wang, Jiahao Lin, Di Zhang, Yipeng Jin, Yongping Bao, Degui Lin

**Affiliations:** ^1^Department of Veterinary Clinical Science, College of Veterinary Medicine, China Agricultural University, Beijing, China; ^2^Faculty of Medicine and Health, Norwich Medical School, University of East Anglia, Norwich, United Kingdom

**Keywords:** BITC, apoptosis, cyclin B1, canine mammary tumor, Cdk1

## Abstract

**Background:** Canine mammary carcinoma is common in female dogs, and its poor prognosis remains a serious clinical challenge, especially in developing countries. Benzyl isothiocyanate (BITC) has attracted great interest because of its inhibitory effect against tumor activity. However, its effect and the underlying mechanisms of action in canine mammary cancer are not well-understood. Here, we show that BITC suppresses mammary tumor growth, both *in vivo* and *in vitro*, and reveal some of the potential mechanisms involved.

**Methods:** The effect of BITC on canine mammary cancer was evaluated on CIPp and CMT-7364, canine mammary carcinoma lines. The cell lines were treated with BITC and then subjected to wound healing and invasion assays. Cell cycles and apoptosis were measured using flow cytometry; TUNEL assay; immunohistochemistry (IHC) for caspase 3, caspase 9, and cyclin D1; hematoxylin and eosin (H&E) staining; and/or quantitative polymerase chain reaction (qPCR).

**Results:** BITC showed a strong suppressive effect in both CIPp and CMT-7364 cells by inhibiting cell growth *in vitro*; these effects were both dose- and time-dependent. BITC also inhibited migration and invasion of CIPp and CMT-7364 cells. BITC induced G2 arrest and apoptosis, decreasing tumor growth in nude mice by downregulation of cyclin B1 and Cdk1 expression.

**Conclusion:** BITC suppressed both invasion and migration of CIPp and CMT-7364 cells and induced apoptosis. BITC inhibited canine mammary tumor growth by suppressing cyclinB1 and Cdk1 expression in nude mice.

## Introduction

Mammary gland carcinomas are the most common cancers in women and intact female dogs (1). Breast cancer is a growing cause of cancer-related deaths among women worldwide, despite targeted therapy and advanced techniques enabling early detection of the disease (2). Canine mammary tumors (CMTs) share similar molecular, epidemiological, and biological features with breast cancer in humans, and these characteristics have been proven to be useful in understanding complex molecular aspects of human tumors (3). There is increasing interest in immunotherapeutic strategies to treat cancers, such as chimeric antigen receptor (CAR) T-cell therapy (4) and PDL-1 antibody drugs (5). The dog is an excellent animal model for testing these modalities in preclinical studies because of the remarkable similarities with humans. There is an increasing need for novel potential antitumor chemical agents to inhibit the growth of CMT and prolong patient survival.

Benzyl isothiocyanate (BITC) is a member of the isothiocyanate (ITC) family that occurs naturally in cruciferous vegetables such as cauliflower and cabbage (6). BITC has been reported to process strong anticancer properties (7). Many studies have indicated that BITC prevents cancer via various chemopreventive pathways, and effects such as the induction of apoptosis in cancer cells are also involved (8). *In vitro* studies have revealed that BITC plays an essential role in preventing cancer by inhibiting cyclin B1/Cdk1 complex expression, which results in cell cycle arrest (9). The G2 transition in the cell cycle requires an active cyclin B1/Cdk1 complex (10). Moreover, BITC has also been reported to exhibit anticancer effects *in vivo* via the inhibition of chemically induced cancer (11).

In the present study, we investigated the effects and mechanism by which BITC inhibits the proliferation of CMT cells *in vitro*. BITC was found to induce apoptosis to suppress tumor growth in nude mice by downregulating cyclin B1 and Cdk1 expression. This study reveals the effect of BITC in inhibiting mammary cancer and indicates its potential for prevention or treatment of mammary cancer in both dogs and humans.

## Methods

### Cell Lines and Cell Culture

The CIPp cell line is a CMT cell line that was kindly provided by the Graduate School of Agricultural and Life Sciences, University of Tokyo (12). The CMT-7364 cell line, another CMT cell line, was isolated and generated in our laboratory (13). Both cell lines were cultured in DMEM (Gibco #C11995500BT) medium supplemented with 10% fetal bovine serum (Gibco #16000044) and incubated under 5% CO_2_ at 37°C.

### Reagents, Antibodies, and Mice

Dimethylsulfoxide (DMSO) (#D2650) and BITC (#252492) were bought from Sigma, and 4,6-diamidino-2-phenylindole (DAPI) (Invitrogen #D1306) was used at a 1:500 dilution to stain the nuclei.

The following primary antibodies used were purchased from Abcam: a rabbit polyclonal antibody against Caspase 3 (#ab13847), a rabbit monoclonal antibody (EPR17060) against Cyclin B1 (#ab181593), a rabbit monoclonal antibody (EPR18107) against Caspase 9 (#ab202068), a mouse monoclonal (7F7AB10) antibody against AIF (#ab110327), a rabbit monoclonal antibody (EPR165) against Cdk1 (#ab224269), and a rabbit polyclonal antibody against cytochrome (#ab90529). Rabbit monoclonal antibody (EPR165) against Cdk1 and rabbit polyclonal antibody against Caspase 3 (#ab13847) were used at a 1:200 dilution for immunohistochemistry (IHC) with an HRP-labeled anti-rabbit IgG (#G-21231) (1:500 for IHC) secondary antibody purchased from Invitrogen. Rabbit polyclonal antibody against Caspase 3 (#ab13847), rabbit monoclonal antibody (EPR17060) against Cyclin B1 (#ab181593), rabbit monoclonal antibody (EPR18107) against Caspase 9 (#ab202068), mouse monoclonal antibody (7F7AB10) against AIF (#ab110327), and rabbit polyclonal antibody against cytochrome (#ab90529) were used at a 1:500 dilution for immunofluorescence. Appropriate fluorescent secondary antibodies Alexa Fluor 488 (#A-11008 and #A-31561) and 594 (#A32740), also purchased from Invitrogen, were used at a 1:500 dilution.

Female BALB/c nude mice, 4–6 weeks old, were purchased from Beijing Vital River Laboratory Animal Technology and used for tumor xenografts. All animal procedures were approved by the Institutional Animal Care and Use Committee of China Agricultural University (approval number: AW20078102-2) in accordance with the Chinese guidelines for the care and use of laboratory animals.

### Evaluation of Cell Proliferation

CMT-7364 and CIPp cells were seeded in 96-well plates at a concentration of 1 × 10^4^ cells per well and incubated for 12 h in 5% CO_2_ at 37°C. Cells were treated with different concentrations of BITC (6.25, 12.5, 25, and 50 μM) dissolved in solvent (DMSO) or solvent alone as a control. After 12, 24, 36, or 48 h, cell viability was evaluated with Cell Counting Kit-8 (Solarbio #CK04-500T) according to the manufacturer's instructions. The optical density (OD) was measured with a microplate reader (BioTek Instruments, Inc., USA) at 450 nm.

For the colony formation assay, CMT-7364 and CIPp cells were grown in six-well plates at 1,000 cells per well, and then treated with different concentrations of BITC (2.5, 5, and 10 μM) or with solvent alone as a control. After 24, 48, or 72 h of treatment, the wells were washed with PBS (Hyclone #SH30256) and incubated with DMEM supplemented with 10% FBS. After incubation for 10 days, the cells were washed twice with PBS and stained with 0.1% (w/v) crystal violet (Solarbio #G1063). The stained cells were imaged using a bright-field microscope (Zeiss).

### Wound Healing Assay

CMT-7364 and CIPp cells were plated in six-well plates at a density of 5 × 10^5^ cells per well and grown to confluence. Then, the monolayer cells were scratched with a 200 μl pipette tip to create a 0.4-mm-wide wound. Cells were treated with different concentrations of BITC (2.5, 5, and 10 μM) or solvent alone as a control in FBS-free DMEM and then incubated at 37°C for 12, 24, and 36 h. Then, plates were observed and photographed using a microscope (Olympus Corporation, Japan). The relative areas of open wounds were analyzed using Image Pro Plus 7 (Media Cybernetics, Inc., Rockville, MD, USA).

### Invasion Assay

Transwell filters (8 μm pore size, Corning #3495) were placed in 24-well plates. Then, the CMT-7364 and CIPp cells were seeded onto the filters at a density of 2,000 cells per chamber in 100 μl of FBS-free DMEM with different concentrations of BITC (2.5, 5, and 10 μM) or solvent alone as a control. The lower chambers were filled with 500 μl of DMEM with 10% FBS. After incubating for 24 h at 37°C, the cells on the top side of the filters were removed with a tipped swab. The cells that had migrated to the lower chamber were stained with 0.1% (w/v) crystal violet and observed using a microscope (Olympus Corporation, Japan).

### Flow Cytometry

CIPp cells were seeded in six-well plates at 2 × 10^5^ cells per well and treated with different concentrations of BITC (0, 0.625, 1.25, 2.5, 5, or 10 μM). Cells were detached using 0.25% trypsin (Gibco #25200072). Cells were collected and washed with PBS three times and then resuspended in 400 μl of binding buffer (BD Biosciences #556454). Cell cycle fractions were determined using PI/RNase staining buffer (BD Biosciences #550825), and cell apoptosis was analyzed using the Annexin V-FITC/PI apoptosis detection kit (BD Biosciences #556419). All samples were collected and analyzed on a FACS Calibur flow cytometer (BD Biosciences, USA), and data were analyzed with Flow Jo software (Version 7.6.1, USA).

### Mouse Xenografts

Tumor xenografts were established in female 4- to 6-week-old BALB/c nude mice by subcutaneous injection of 2 × 10^6^ CIPp cells suspended in 100 μl of PBS into the mammary fat pad. On the seventh day after inoculation, six mice from each group were administered BITC (20 mg/kg body weight) or solvent alone (control) daily via intraperitoneal injections. Tumor growth (tumor length and width) and body weights were measured every 2 days until the 21st day post-injection. Mice were humanely euthanized via cervical dislocation under isoflurane anesthesia, and the tumors were harvested. CIPp xenograft tumor volumes were measured and then fixed with 10% (v/v) neutral-buffered formalin (Solarbio #G2161), embedded in paraffin wax, and sectioned serially at a thickness of 3 μm. Tumor volume was calculated using the formula length × width^2^/2.

A TUNEL technology kit (Roche Applied Science #APT110) was used for analysis of the tumor tissue (control and BITC groups). The paraffin-embedded tissue was pretreated, and the tissue sections were dewaxed and rehydrated. The tissue sections were then incubated with proteinase K, fixed with a paraformaldehyde solution, and permeabilized in a sodium citrate solution. All samples were labeled with the TUNEL reaction mixture (TdT enzyme solution and FITC solution). The tissues were analyzed under a fluorescent microscope (Olympus Corporation, Japan) using an excitation wavelength of 530 nm and a detection wavelength of 630 nm. The data were quantified by ImageJ software (Rasband, W.S., ImageJ, National Institutes of Health, Bethesda, MD, USA) with the following macro (14).

For IHC, deparaffinized and rehydrated sections were subjected to microwave-based antigen retrieval in citrate buffer (~0.24% trisodium citrate dihydrate, ~0.038% citric acid, in water). The sections were then incubated in hydrogen peroxide buffer (10% H_2_O_2_ in methanol) to mask any background peroxidase activity followed by treatment with blocking solution (10% goat serum, 0.3% Triton X-100 in PBS). Sections were stained with appropriate primary antibodies (Caspase 3, caspase 9, AIF, cytochrome C, Cdk1, and Cyclin B1 antibodies) and secondary antibodies (anti-rabbit HRP, anti-rabbit Alexa Fluor 488, anti-mouse Alexa Fluor 488, and anti-rabbit Alexa Fluor 594 antibodies). The signals were developed with diaminobenzidine buffer, and then the sections were counter-stained with hematoxylin. Tissue sections were mounted using cover glass and mounting medium (Neomount; Merck, 1,090,160,100) and imaged using 20× and 40× objectives on a bright-field microscope (Zeiss). Images were captured with a digital microscope, and the amounts of Caspase 3/Cyclin B1–positive cells and total cells per image were automatically calculated by color using ImageJ software (Rasband, W.S., ImageJ, National Institutes of Health, Bethesda, MD, USA). The ratio between Caspase 3/Cyclin B1–positive cells and total cells was defined as the percentage of Caspase 3/Cyclin B1–positive cells.

Total RNAs (some parts of the xenografts that were frozen) were isolated using TRIzol reagent (Invitrogen #15596018) according to the manufacturer's instructions. The RNA was reverse-transcribed into cDNA using a Revert Aid First Strand cDNA Synthesis Kit (ThermoFisher #K1622). The primers used for amplifying Bax and Bcl-2 were designed using Primer 5.0 software, and the primer sequences are shown in Table 1. Quantitative polymerase chain reaction (qPCR) was performed using a 7500 Fast Real-Time PCR System (Applied Biosystems, USA) with SYBR green. Each sample was subjected to three repetitions. Results were expressed using the 2^−ΔΔCt^ comparative method: ΔΔCt = (target gene Ct of experimental group – reference gene Ct of experimental group) – (target gene Ct of control group – reference gene Ct of control group). *Beta-actin* was used as the reference gene.

**Table 1:** Sequences of primers used for quantitative polymerase chain reaction (qPCR).

**Gene**	**Sequence (5^**′**^-3^**′**^): sense and antisense**	**Product size (bp)**
*Bcl2*	Sense: 5′-TGGGATGCCTTTGTGGAACTG-3′ Antisense: 5′-TCTTCAGAGACAGCCAGGAGAA-3′	73
*Bax*	Sense: 5′-GACGAACTGGACAGTAACATGGAGCT-3′ Antisense: 5′-GGCAAAGTAGAAAAGGGCGACAAC-3′	150
*Beta-actin*	Sense: 5′-ACTTAGTTGCGTTACACCCTT-3′ Antisense: 5′- GTCACCTTCACCGTTCCA-3′	156

### Statistical Analysis

All data are presented as mean or mean ± standard deviation (mean ± SD) for three independent experiments. We analyzed the differences in data between BITC treatment and control by *t*-tests or two-way ANOVA (Primer GraphPad 5 software, USA). Differences were considered significant at *P* < 0.05, <0.01, and <0.001, denoted by *, **, and ***, respectively.

## Results

### Cell Proliferation Decreased Significantly After Treatment With BITC

**Figure 1:**
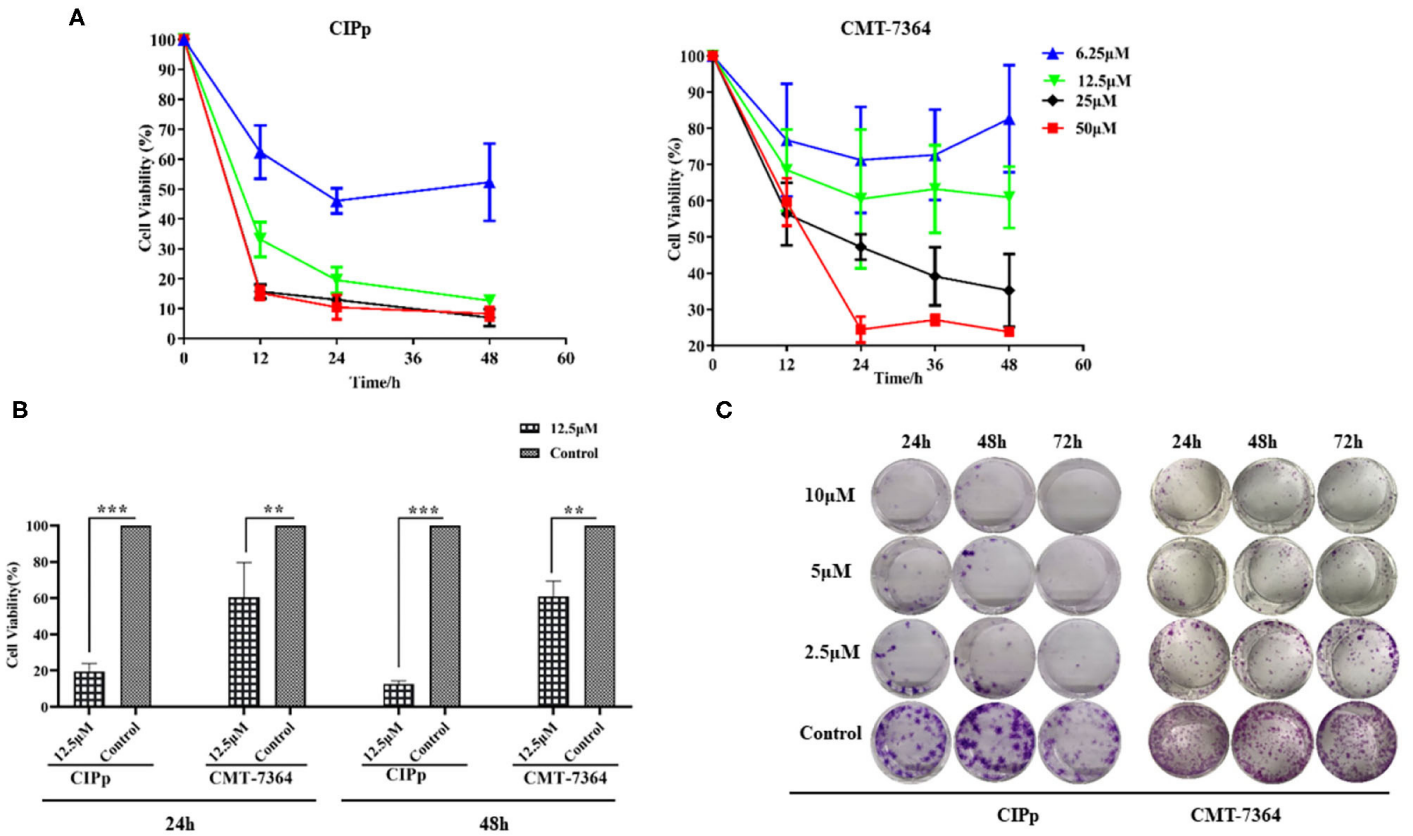
**(A)** Cell viability was analyzed using Cell Counting Kit-8 after 0, 12, 24, 36, and 48 h following benzyl isothiocyanate (BITC) treatment, at various concentrations, in CIPp cells and CMT7364 cells. **(B)** Cell viability was detected 24 and 48 h after treatment with 12.5 μM BITC and normalized to that of controls. **(C)** Colony formation of CIPp cells and CMT7364 cells. Cells were treated with 10, 5, and 2.5 μM BITC for 24, 48, and 72 h; this was followed with 0.1% (w/v) crystal violet staining of attached cells after 10 days. **p < 0.01, ***p < 0.001.

CIPp cells and CMT-7364 cells were treated with various doses of BITC. Cell viability decreased with increasing doses of BITC (Figure 1A). The viability of CIPp and CMT-7364 cells at 24 and 48 h following treatment with 12.5 μM of BITC was significantly decreased compared with that of the control (Figure 1B). At both time points, a significant decrease in cell viability relative to the controls was observed. The ability of CIPp cells and CMT-7364 cells to form colonies was evaluated following treatment with 2.5, 5, and 10 μM of BITC (Figure 1C). The results of 0.1% (w/v) crystal violet staining suggested that BITC significantly inhibited colony formation.

### BITC Inhibited Cell Migration and Invasion

**Figure 2:**
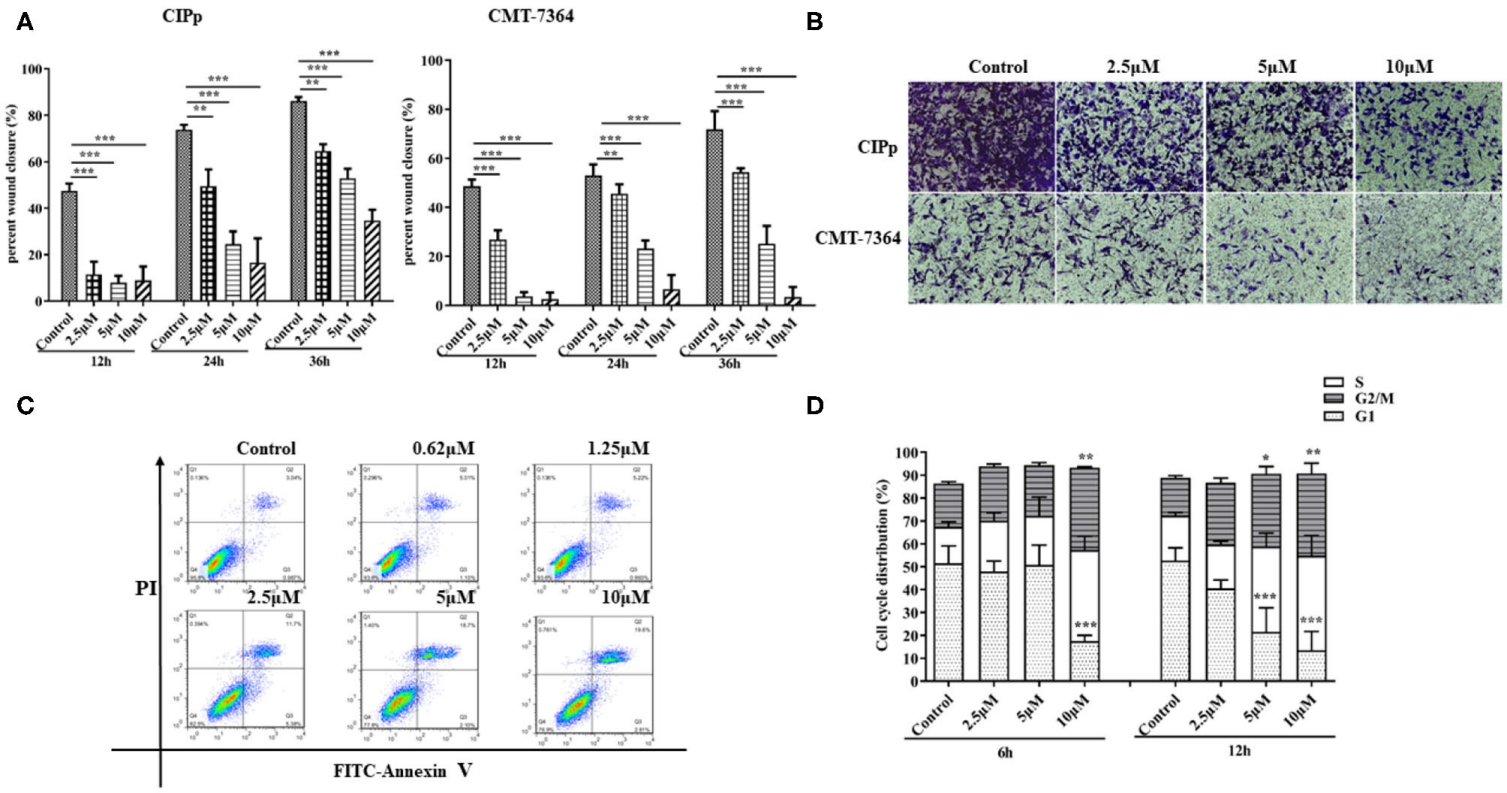
**(A)** The migratory ability of CIPp and CMT7364 cells was tested at 12, 24, and 36 h post-treatment. **(B)** Invasion assay: CIPp and CMT7364 cells were treated with various concentrations of BITC and stained with 0.1% (w/v) crystal violet after 10 days. Flow cytometry **(C,D)**. **(C)** Flow cytometric analysis of Annexin V/PI staining in cells. CIPp cells were treated with 0.62, 1.25, 2.5, 5, or 10 μM BITC for 12 h. **(D)** CIPp cells were collected after 6 and 12 h of treatment, and data were analyzed. *p < 0.05, **p < 0.01, ***p < 0.001.

The suppressive effect of BITC on cell migration and invasion was evaluated on both CIPp cells and CMT-7364 cells by subjecting them to BITC treatment or not. A significantly higher inhibitory effect was observed in cells treated with 10 μM BITC for 12, 24, and 36 h, compared with that in non-treated cells. The wounds were almost completely healed (86.09 ± 1.76% in CIPp cells, 71.83 ± 7.38% in CMT-7364, respectively) in the control group, while the BITC groups showed a decrease in healing to 34.77 ± 4.59% in CIPp cells and 3.53 ± 1.01% in CMT-7364 cells at 36 h post-treatment (Table 2 and Figure 2A). BITC caused a reduction in the migration of the cells into the wound areas in both cell lines. Transwell assay results showed that the inhibitory effect of BITC on the invasion of CIPp cells and CMT-7364 cells was dose-dependent (Figure 2B). Compared with that in the BITC groups, more cells were seen in the lower chambers in control groups.

**Table 2:** Results of wound healing assay (%).

**Cell line**	**Post-treatment (h)**	**Control**	**2.5 μM BITC**	**5 μM BITC**	**10 μM BITC**
CMT7364	12	48.70 ± 2.64	26.92 ± 3.71^***^	3.69 ± 1.76^***^	2.66 ± 2.63^***^
	24	52.99 ± 4.53	45.56 ± 3.87^**^	23.17 ± 3.3^***^	6.67 ± 5.79^***^
	36	71.83 ± 7.38	54.43 ± 1.50^**^	25.21 ± 7.24^***^	3.53 ± 1.01^***^
CIPp	12	47.43 ± 3.14	11.45 ± 5.44^***^	7.92 ± 2.90****	8.89 ± 5.95^***^
	24	73.8 ± 2.00	49.51 ± 7.16^**^	24.51 ± 5.42^***^	16.56 ± 10.42^***^
	36	86.09 ± 1.76	64.61 ± 2.90^***^	52.85 ± 4.11^***^	34.77 ± 4.59^***^

*BITC, benzyl isothiocyanate*.

** *p < 0.01*,

*** *p < 0.001*.

### BITC Induced Cell Cycle Arrest at the G2 Phase and Apoptosis

To determine whether BITC inhibits CMT cell proliferation through the induction of apoptosis, the apoptotic rates were measured by Annexin V/PI staining. There was an increase in apoptosis rate in the CIPp cell line after 10 μM BITC treatment for 24 h (Figure 2C), indicating that BITC inhibited mammary cancer cell viability by inducing apoptosis. Cell cycle progression was evaluated via flow cytometry in the CIPp cell line. BITC treatment increased the percentage of cells in the G2 phase in the cell line at 6 and 12 h post-treatment (Figure 2D) (*p* < 0.05). Distribution of cells in the G2 phase was dose-dependent: higher concentrations of BITC enhanced cell cycle arrest at the G2 phase.

### BITC Suppressed CIPp Xenograft Tumor Growth *in vivo*

**Figure 3:**
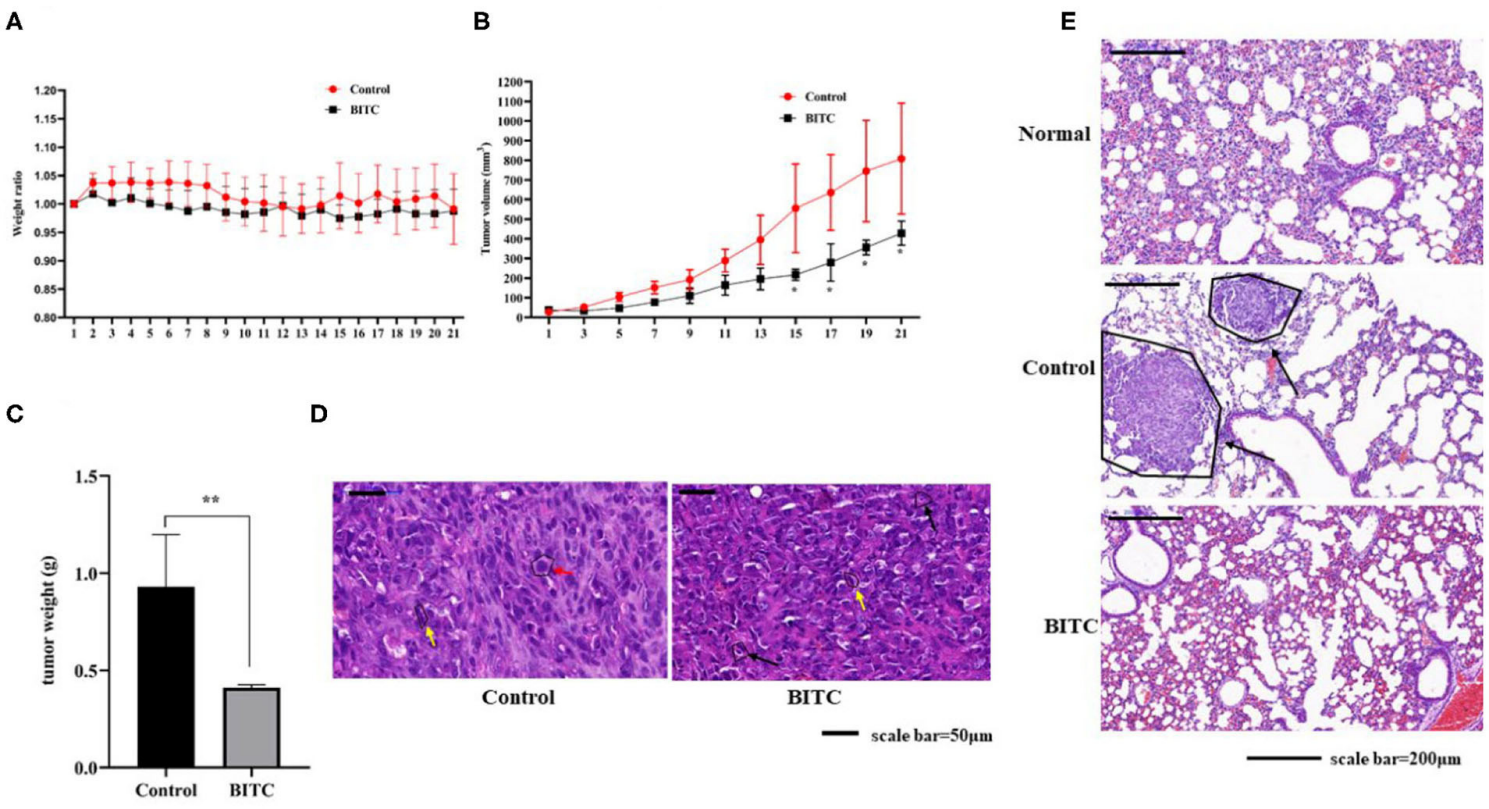
BITC inhibits CIPp tumor growth *in vivo*. **(A)** Mouse body weight ratio throughout the treatment. **(B)** Tumor volume. **(C)** Tumor weight at day 21. **(D)** hematoxylin and eosin (H&E)–stained tumor tissues (scale bar=50 μm). Red arrow: karyomegaly. Yellow arrow: mitosis. Black arrow: apoptosis. **(E)** The H&E-stained lung tissues of mice. “Normal” represents the lungs of healthy mice (scale bar= 200 μm). Black arrow: lung metastasis. **p < 0.01.

As mentioned above, BITC inhibited CMT cell proliferation *in vitro*. Therefore, its ability to suppress tumor growth *in vivo* was further investigated. The mice in both control and BITC groups had similar body weights (Figure 3A) and similar serum levels of alanine aminotransferase (ALT), aspartate aminotransferase (AST), alkaline phosphatase (ALP), gamma-glutamyl transpeptidase (GGT), total bilirubin (TBIL), blood urea nitrogen (BUN), and creatinine (CRE), indicating that there were no differences in terms of organ damage between the two groups at 21 days post-xenograft (Supplementary Table 1 and Supplementary Figure 1). However, the tumor volumes and weights of the control group were significantly higher than those of the tumors isolated from the BITC group at the end of the treatment period (Figures 3B,C). Microscopic analysis of hematoxylin and eosin (H&E)–stained tumor sections revealed typical tumor histological features. The nuclei were generally larger than those of normal cells (see red arrow in Figure 3D), and mitotic figures were found (yellow arrow). Additionally, there were several apoptotic cells in the BITC group (black arrow). We also found metastatic tumors in the lungs of the control group (displayed black arrow in Figure 3E). Tumor-induced inflammation was observed in both the BITC and control groups but not in the lungs of healthy mice.

### BITC Induced Tumor Apoptosis *in vivo*

**Figure 4:**
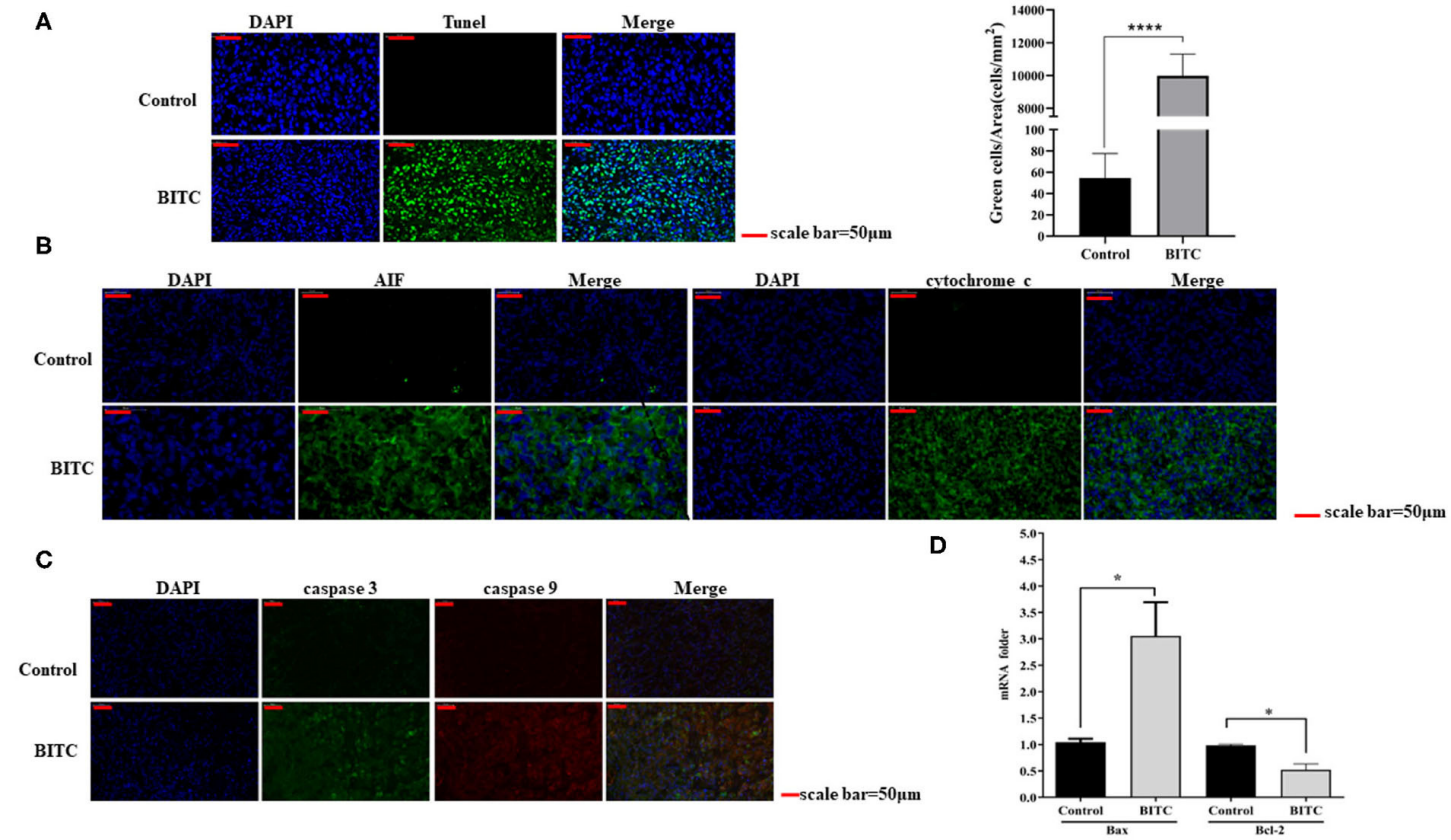
**(A)** TUNEL staining of tumor tissue (scale bar = 50 μm). The apoptotic cells were stained by the TUNEL method representing green fluorescence. **(B)** AIF and cytochrome c expressed in tumor tissue. The green fluorescence represents the expression of AIF and cytochrome c. **(C)** Immunofluorescence images showing the expression of caspase 3 and caspase 9. The green fluorescence represents the expression of caspase 3, and the red fluorescence represents the expression of caspase 9. **(D)** Bax/Bcl-2 mRNA expression in tumor tissue; mRNA levels were measured by quantitative polymerase chain reaction (qPCR). *p < 0.05, ****p < 0.001.

In this study, tumor tissues from BITC-treated mice showed a higher level of apoptosis than those of the control group (Figure 4A). Total RNA was extracted from the tumor tissue, and the mRNA levels of Bax and Bcl-2 were evaluated by qPCR: in the BITC groups, higher expression of Bax and lower expression of Bcl-2 were observed (Figure 4D). We observed high expression of caspase 3 (the inactive pro caspase 3 and cleaved caspase 3) and caspase 9, members of the caspase family of protease enzymes that play key roles in programmed cell death, including apoptosis (Figure 4C). AIF and cytochrome c, which are important caspase factors, were also expressed at high levels during apoptosis (Figure 4B). These results indicate that BITC induces apoptosis of tumor cells and suppresses tumor growth *in vivo*.

### BITC Regulated the Expression of Cdk1 and Cyclin B1 *in vivo*

**Figure 5:**
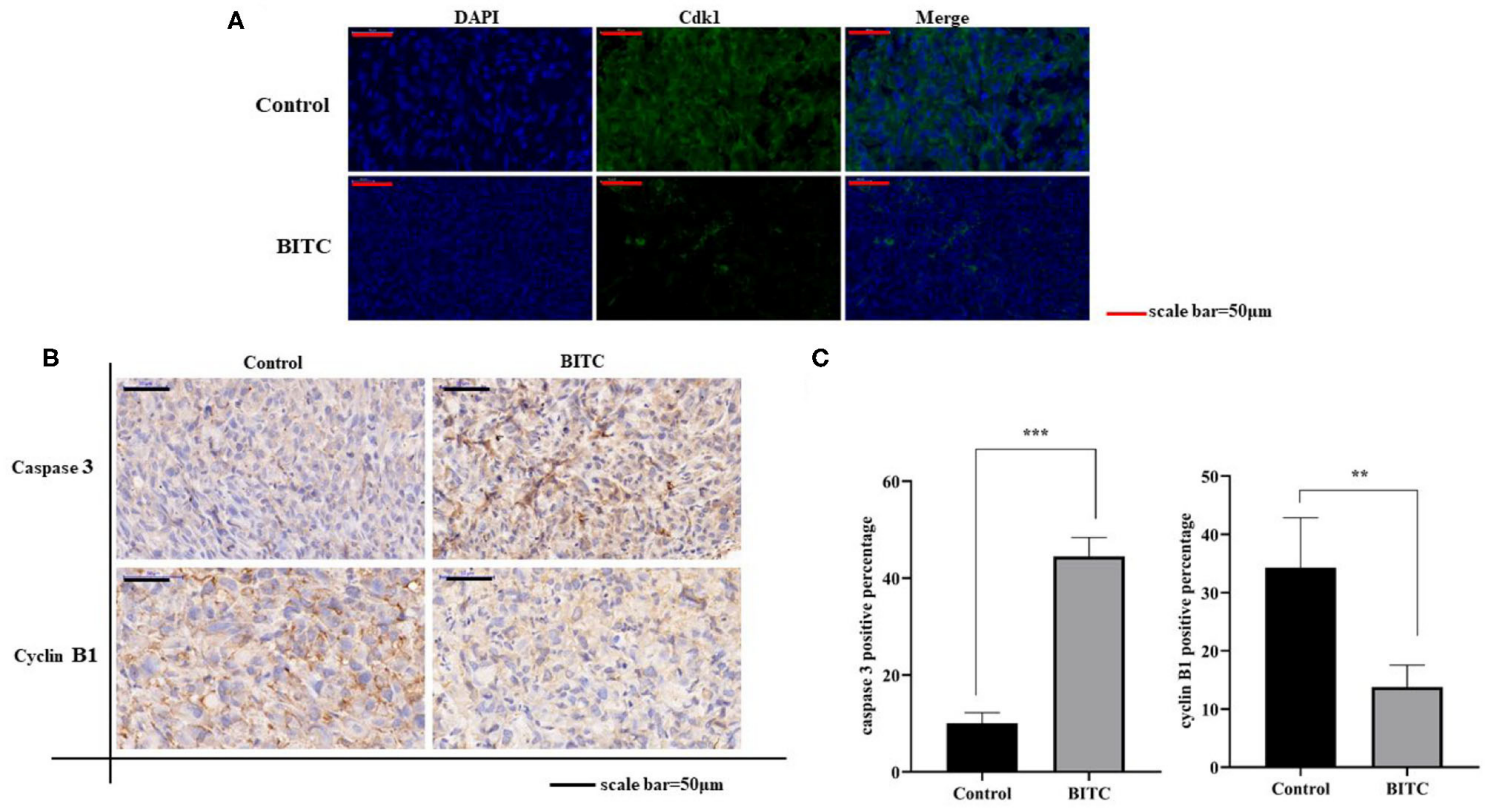
**(A)** Cdk1 expressed in tumor tissue (scale bar = 50 μm). The green fluorescence represents the expression of Cdk1. **(B)** Immunohistochemistry (IHC) images (scale bar = 50 μm) of tumor tissue expressing caspase 3 and cyclin B1. **(C)** Images of caspase 3 and cyclin B1 staining (scale bar = 50 μm). **p < 0.01, ***p < 0.001.

To further investigate the mechanisms by which BITC suppresses tumor growth, cell cycle distribution was measured *in vitro*. The results showed that more cells were arrested in the G2 phase after treatment compared with the untreated controls (Figure 2D). Proteins that regulate cell cycle progression, such as Cdk1 and cyclin B1, were also observed *in vivo*. The expression of Cdk1 protein in the BITC groups was lower than in the control groups (Figure 5A) during apoptosis, and a similar result was observed in terms of the expression of cyclin B1 (Figures 5B,C). Thus, BITC reduced cyclin B1 and Cdk1 levels, inducing apoptosis to suppress tumor growth.

## Discussion

In this study, BITC showed significant anti-proliferative effects in two CMT cell lines (Figure 1A). A recent study reported a similar effect of BITC on the viability of an oral cancer cell line (7) and additionally demonstrated that BITC inhibited cell colony formation in a time- and dose-dependent manner. A significant difference in the migration and invasion ability of the two cell lines was observed post–BITC treatment. After the BITC treatment, MCT-7364, an epithelium-like cell line, was more sensitive to BITC treatment than CIPp cells according to the wound healing assay results (Figure 2A); this may be due to the lack of expression of E-cadherin (15), a gene that mediates cell-to-cell adhesion and is deficient in MCT-7364 cell lines (13). MCT-7364 is a triple-negative canine mammary cancer cell line that was established and characterized by Zhang et al. (13). Tumor tissue was found to be negative for HER-2 by IHC analysis; in comparison, CIPp is a HER-2–positive canine mammary cancer cell line (12). There are four major molecular subtypes of breast cancer: luminal A, luminal B, triple-negative, and HER2-enriched (16). There are differences between two subtypes of breast cancers: triple-negative canine mammary cancer cell lines are negative for estrogen receptor (ER), progesterone receptor (PR), and HER-2, while HER-2 enriched cell lines are generally HER-2–positive and lymph node–positive (17, 18). Some drugs or chemicals exert anticancer effects by altering cell cycle progression. In canine mammary carcinoma, ivermectin induces cell cycle arrest at the G1 phase (19). BITC can also induce cell cycle arrest but mostly at the G2 phase (8). In order to investigate the effect of BITC on tumor growth *in vivo*, tumor cell injection was carried out in nude mice. A decrease in both weights and volumes of mammary metastases was observed following BITC treatment. Tumors were observed in the lungs of the control group but not in those of mice subjected to BITC treatment. This indicates that BITC inhibits xenografts and suppresses tumor growth (Figure 3B). Moreover, high doses of BITC treatment did not produce any changes in the serological levels of ALT, AST, ALP, GGT, TBIL, BUN, and CRE, indicating that the administration of BITC did not cause any unexpected organ damage (Supplementary Table 1 and Supplementary Figure 1). Together, these data indicate that BITC is a potential natural anticancer agent that is safe to combine with other therapies for treating mammary cancer (20). Some recent studies have focused on nanoparticles of sulforaphane, an extensive ITC, and its anticancer effect has been confirmed both *in vivo* and *in vitro* (21, 22). Those findings suggest the potential to synthesize BITC nanoparticles with enhanced anticancer effects to act as a potential tool for BITC delivery.

This study revealed the mechanisms underlying the anticancer activity of BITC *in vivo*. TUNEL assay results revealed marked apoptosis (Figure 4A). During this process, the expression of apoptosis factors, such as AIF and cytochrome c, was upregulated (Figure 4B); moreover, the level of Bax expression in the control group was lower than in the BITC-treated group. However, in contrast, the expression of Bcl-2, a gene that suppresses apoptosis (23), was significantly lower in the BITC-treated group (Figure 4D). The Bcl-2 protein belonging to the Bcl-2 family, which is a family of proteins regulating apoptosis, is often overexpressed in many tumors (24). In addition, the expression of cyclin B1/Cdk1 decreased after BITC treatment (Figures 5A–C); it is well-known that cell cycle progression is controlled by various cyclins and cyclin-dependent kinases (Cdks) (25). The expression of Cdk1 and cyclin B1 is required for the transition from G2 phase into mitosis (26). Here, the levels of both cyclin B1 and Cdk1 were reduced. In the present study, BITC inhibited the migration and invasion of CIPp and CMT-7364 cells and induced apoptosis. The expression of Cyclin B1 and CDK1 was reduced in tumors from BITC-treated mice, which resulted in the suppression of tumor growth. Therefore, these data reveal that BITC possesses anticancer activity and therapeutic potential for mammary cancer in both dogs and humans.

## Data Availability Statement

The original contributions presented in the study are included in the article/Supplementary Material, further inquiries can be directed to the corresponding author/s.

## Ethics Statement

All animal procedures were approved by the Institutional Animal Care and Use Committee of China Agricultural University (approval number: AW20078102-2) in accordance with the Chinese guidelines for the care and use of laboratory animals.

## Author Contributions

NC carried out all the assays of BITC on canine mammary tumor cells and drafted the manuscript. JG, WZ, and QW collected and analyzed the data. HD, ZL, and YZ performed the animal experiment. JL and DZ helped edit the manuscript. YJ, YB, and DL conceived of the study and supervised in its design and coordination. All authors read and approved this final manuscript.

## Conflict of Interest

The authors declare that the research was conducted in the absence of any commercial or financial relationships that could be construed as a potential conflict of interest.

## References

[1] Sorenmo KU Rasotto R Zappulli V Goldschmidt MH. Development, anatomy, histology, lymphatic drainage, clinical features, and cell differentiation markers of canine mammary gland neoplasms. Vet Pathol. (2011) 48:85–97. 10.1177/0300985810389480 21147765

[2] Torre LA Islami F Siegel RL Ward EM Jemal A. Global cancer in women: burden and trends. Cancer Epidemiol Biomarkers Prev. (2017) 26:444–57. 10.1158/1055-9965.EPI-16-0858 28223433

[3] Owen LN. A comparative study of canine and human breast cancer. Invest Cell Pathol. (1979) 2:257–75. 396282

[4] Kato D Yaguchi T Iwata T Morii K Nakagawa T Nishimura R. Prospects for personalized combination immunotherapy for solid tumors based on adoptive cell therapies and immune checkpoint blockade therapies. Nihon Rinsho Meneki Gakkai Kaishi. (2017) 40:68–77. 10.2177/jsci.40.68 28539557

[5] Gou L Gao J Yang H Gao C. The landscape of CAR T-cell therapy in the United States and China: a comparative analysis. Int J Cancer. (2019) 144:2043–50. 10.1002/ijc.31924 30307029

[6] Kim SH Nagalingam A Saxena NK Singh SV Sharma D. Benzyl isothiocyanate inhibits oncogenic actions of leptin in human breast cancer cells by suppressing activation of signal transducer and activator of transcription 3. Carcinogenesis. (2011) 32:359–67. 10.1093/carcin/bgq267 21163886 PMC3105585

[7] Lee CF Chiang NN Lu YH Huang YS Yang JS Tsai SC. Benzyl isothiocyanate (BITC) triggers mitochondria-mediated apoptotic machinery in human cisplatin-resistant oral cancer CAR cells. Biomedicine. (2018) 8:15. 10.1051/bmdcn/2018080315 30141402 PMC6108226

[8] Kalkunte S Swamy N Dizon DS Brard L. Benzyl isothiocyanate (BITC) induces apoptosis in ovarian cancer cells *in vitro*. J Exp Ther Oncol. (2006) 5:287–300. 17024969

[9] Zhou T Li G Cao B Liu L Cheng Q Kong H. Downregulation of Mcl-1 through inhibition of translation contributes to benzyl isothiocyanate-induced cell cycle arrest and apoptosis in human leukemia cells. Cell Death Dis. (2013) 4:e515. 10.1038/cddis.2013.41 23449451 PMC3734843

[10] Nomura N Nomura M Newcomb EW Zagzag D. Geldanamycin induces G2 arrest in U87MG glioblastoma cells through downregulation of Cdc2 and cyclin B1. Biochem Pharmacol. (2007) 73:1528–36. 10.1016/j.bcp.2007.01.022 17324379

[11] Conaway CC Yang YM Chung FL. Isothiocyanates as cancer chemopreventive agents: their biological activities and metabolism in rodents and humans. Curr Drug Metab. (2002) 3:233–55. 10.2174/1389200023337496 12083319

[12] Uyama R Nakagawa T Hong SH Mochizuki M Nishimura R Sasaki N. Establishment of four pairs of canine mammary tumour cell lines derived from primary and metastatic origin and their E-cadherin expression. Vet Comp Oncol. (2006) 4:104–13. 10.1111/j.1476-5810.2006.00098.x 19754820

[13] Zhang H Pei S Zhou B Wang H Du H Zhang D. Establishment and characterization of a new triple-negative canine mammary cancer cell line. Tissue Cell. (2018) 54:10–9. 10.1016/j.tice.2018.07.003 30309498

[14] Maidana DE Tsoka P Tian B Dib B Matsumoto H Kataoka K. A novel ImageJ macro for automated cell death quantitation in the retina. Invest Ophthalmol Vis Sci. (2015) 56:6701–8. 10.1167/iovs.15-17599 26469755 PMC4611955

[15] Canel M Serrels A Frame MC Brunton VG. E-cadherin-integrin crosstalk in cancer invasion and metastasis. J Cell Sci. (2013) 126(Pt 2):393–401. 10.1242/jcs.100115 23525005

[16] Prat A Pineda E Adamo B Galvan P Fernandez A Gaba L. Clinical implications of the intrinsic molecular subtypes of breast cancer. Breast. (2015) 24(Suppl. 2):S26–35. 10.1016/j.breast.2015.07.008 26253814

[17] Voduc KD Cheang MC Tyldesley S Gelmon K Nielsen TO Kennecke H. Breast cancer subtypes and the risk of local and regional relapse. J Clin Oncol. (2010) 28:1684–91. 10.1200/JCO.2009.24.9284 20194857

[18] Metzger-Filho O Sun Z Viale G Price KN Crivellari D Snyder RD. Patterns of Recurrence and outcome according to breast cancer subtypes in lymph node-negative disease: results from international breast cancer study group trials VIII and IX. J Clin Oncol. (2013) 31:3083–90. 10.1200/JCO.2012.46.1574 23897954 PMC3753700

[19] Diao H Cheng N Zhao Y Xu H Dong H Thamm DH. Ivermectin inhibits canine mammary tumor growth by regulating cell cycle progression and WNT signaling. BMC Vet Res. (2019) 15:276. 10.1186/s12917-019-2026-2 31375107 PMC6679554

[20] Hecht SS. Chemoprevention of cancer by isothiocyanates, modifiers of carcinogen metabolism. J Nutr. (1999) 129:768S−74S. 10.1093/jn/129.3.768S 10082787

[21] Soni K Kohli K. Sulforaphane-decorated gold nanoparticle for anti-cancer activity: *in vitro* and *in vivo* studies. Pharm Dev Technol. (2019) 24:427–38. 10.1080/10837450.2018.1507038 30063165

[22] Xu Y Han X Li Y Min H Zhao X Zhang Y. Sulforaphane mediates glutathione depletion via polymeric nanoparticles to restore cisplatin chemosensitivity. ACS Nano. (2019) 13:13445–55. 10.1021/acsnano.9b07032 31670945

[23] Korsmeyer SJ Shutter JR Veis DJ Merry DE Oltvai ZN. Bcl-2/Bax: a rheostat that regulates an anti-oxidant pathway and cell death. Semin Cancer Biol. (1993) 4:327–32. 8142617

[24] Cory S Adams JM. Killing cancer cells by flipping the Bcl-2/Bax switch. Cancer Cell. (2005) 8:5–6. 10.1016/j.ccr.2005.06.012 16023593

[25] Hartwell LH Weinert TA. Checkpoints: controls that ensure the order of cell cycle events. Science. (1989) 246:629–34. 10.1126/science.2683079 2683079

[26] Malumbres M. Cyclin-dependent kinases. Genome Biol. (2014) 15:122. 10.1186/gb4184 25180339 PMC4097832

