# Supplementary material for: Benzyl Isothiocyanate Induces Apoptosis and Inhibits Tumor Growth in Canine Mammary Carcinoma via Downregulation of the Cyclin B1/Cdk1 Pathway
Source: Front Vet Sci. 2020 Nov 11;7:580530. doi: 10.3389/fvets.2020.580530 (PMC7686582; doi:10.3389/fvets.2020.580530)
Supplement: Supplementary file 1 [file Data_Sheet_1.pdf]

Additional file

Table S1 **Serum chemistry of xenotransplant mice**

group	ALT U/L	AST U/L	ALP U/L	GGT U/L	TBIL mg/dL	BUN mg/dL	CRE mg/dL
control	27	89	203	<1.0	0.06	22.83	0.26
	28	141	192	<1.0	0.23	27.41	0.21
	29	142	65	<1.0	0.10	29.46	0.29
	29	154	72	<1.0	<0.02	21.93	0.23
	29	122	70	<1.0	0.09	25.88	0.25
	20	114	52	<1.0	0.04	23.42	0.21
mean ± SD	27±3.52	127±23.61	109±60.00	-	0.10±0.07	25.16±2.93	0.24±0.03
BITC	28	146	55	<1.0	0.04	29.09	0.27
	12	112	50	<1.0	0.07	20.20	0.29
	23	137	80	<1.0	0.09	24.44	0.30
	24	114	70	<1.0	0.10	22.63	0.29
	23	150	48	<1.0	0.07	28.67	0.27
	29	178	112	<1.0	0.10	29.00	0.23
mean ± SD	23.17±6.04	139.50±24.68	69.17±24.36	-	0.07±0.02	25.67±3.80	0.27±0.02

Fig. S1

a

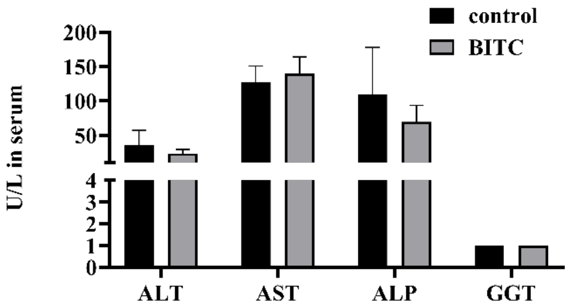

b

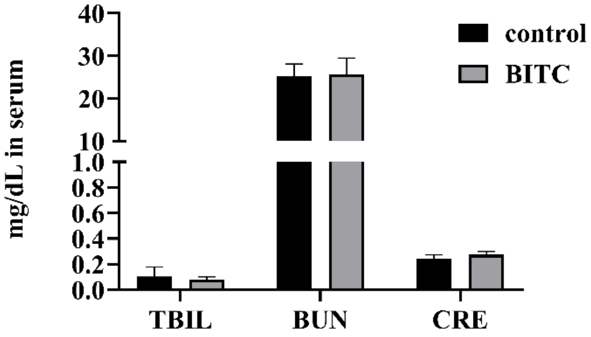

40
30
20
10
1.0
0.8
0.6
0.4
0.2
0.0
mg/dL in serum
control
BITC
TBIL
BUN
CRE
